# Infective Pleural Effusions—A Comprehensive Narrative Review Article

**DOI:** 10.3390/clinpract14030068

**Published:** 2024-05-16

**Authors:** Mohammad Abdulelah, Mohammad Abu Hishmeh

**Affiliations:** 1 Department of Internal Medicine, University of Massachusetts Chan Medical School—Baystate Campus, Springfield, MA 01199, USA; 2 Department of Pulmonary and Critical Care Medicine, University of Massachusetts Chan Medical School—Baystate Campus, Springfield, MA 01199, USA

**Keywords:** pleural infections, infectious pleural effusions, pleural disease, empyema, pneumonia, exudative pleural effusion, parapneumonic effusion, intrapleural enzyme therapy

## Abstract

Infective pleural effusions are mainly represented by parapneumonic effusions and empyema. These conditions are a spectrum of pleural diseases that are commonly encountered and carry significant mortality and morbidity rates reaching upwards of 50%. The causative etiology is usually an underlying bacterial pneumonia with the subsequent seeding of the infectious culprit and inflammatory agents to the pleural space leading to an inflammatory response and fibrin deposition. Radiographical evaluation through a CT scan or ultrasound yields high specificity and sensitivity, with features such as septations or pleural thickening indicating worse outcomes. Although microbiological yields from pleural studies are around 56% only, fluid analysis assists in both diagnosis and prognosis by evaluating pH, glucose, and other biomarkers such as lactate dehydrogenase. Management centers around antibiotic therapy for 2–6 weeks and the drainage of the infected pleural space when the effusion is complicated through tube thoracostomies or surgical intervention. Intrapleural enzymatic therapy, used to increase drainage, significantly decreases treatment failure rates, length of hospital stay, and surgical referrals but carries a risk of pleural hemorrhage. This comprehensive review article aims to define and delineate the progression of parapneumonic effusions and empyema as well as discuss pathophysiology, diagnostic, and treatment modalities with aims of broadening the generalist’s understanding of such complex disease by reviewing the most recent and relevant high-quality evidence.

## 1. Introduction

Pleural effusions are commonly encountered in daily clinical practice with variable, often clinically subtle, presentations. They have been estimated to affect over 1.5 million patients annually in the United States [1]. More than 50 underlying etiologies of pleural effusion are recognized, which contributes to the complexity of such disease. Congestive heart failure is the most frequently encountered etiology, affecting around 500,000 patients annually [2]. Effusions are classified as transudative and exudative effusions based on Light’s criteria, which was established by Dr. Richard Light in 1972 after hypothesizing the etiologies of pleural effusions in his intern year. While routine transudative and uncomplicated exudative effusions often readily resolve with the treatment of the underlying condition, a subset presents as complicated effusions requiring intricate diagnostic and therapeutic interventions. Overall, parapneumonic effusions and empyema are grouped under the umbrella term of infectious pleural effusions [3]. Uncomplicated parapneumonic effusions denote simple exudative effusions associated with pneumonia that often resolve with antibiotic treatment alone. However, a subset of parapneumonic effusions become complicated by the bacterial invasion of the pleural space as evidenced by the formation of septations and other sonographic appearances that will be discussed later in this text. This can lead to frank empyema, characterized by purulent, loculated pleural fluid that frequently necessitates drainage. The transition from uncomplicated parapneumonic effusion to complicated parapneumonic effusions to empyema represents a dynamic continuum [3]. 

Compared to their uncomplicated counterparts, complicated parapneumonic effusions have been associated with significantly higher mortality, longer hospitalizations, increased healthcare costs, and worse patient-reported outcomes [1,4]. 

Those with indwelling pleural catheters, such as patients with recurrent malignant effusions, signify a high-risk group. A recent meta-analysis revealed that those patients carry a 5.7% risk of pleural [5]. The median time to the occurrence of infection is 60 days, perhaps reflecting the importance of post-insertion catheter care, education, and maintenance [6]. 

Pleural effusions and empyema secondary to tuberculosis represent unique and challenging diseases. Their occurrence is rare in developed countries [7,8]. Findings suggestive of such diseases include a pleural fluid lymphocyte/neutrophil ratio of >0.75 in combination with elevated adenosine deaminase [7]. Regardless, their presence requires a longer duration of treatment [7]. Details regarding the specific diagnosis and management of such conditions will not be discussed in this text as they are infrequently encountered and often require multi-disciplinary and specialized care. 

**Figure 1:**
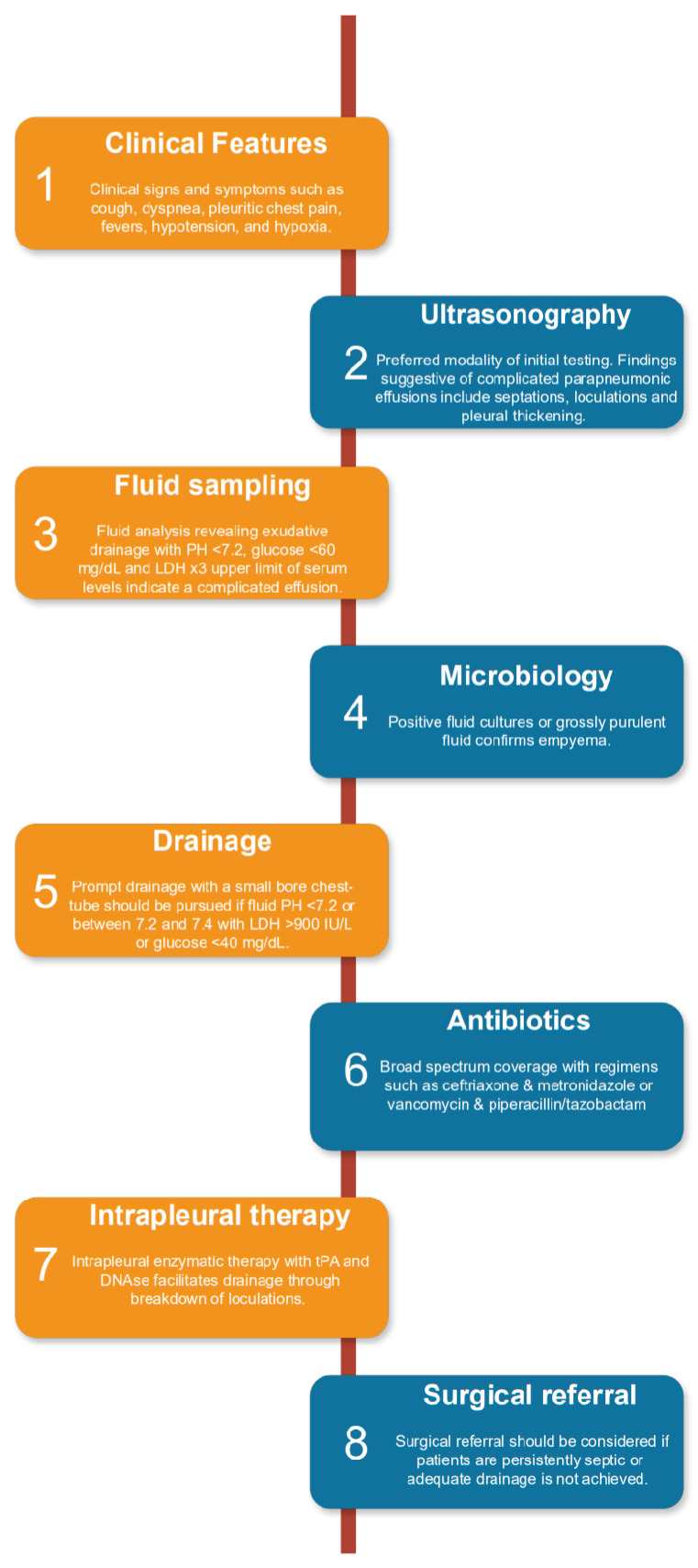
A clinical approach to evaluation of parapneumonic pleural effusions commonly starts with radiographical testing such as chest X-ray, ultrasonography, or computed tomography. Fluid sampling is then pursued, which further guides interventions such as drainage and intrapleural enzymatic therapy. Broad spectrum, parenteral antibiotics should be administered promptly. Adapted from Shen K.R. et al. [9] and Roberts M.E. et al. [10].

The symptoms observed in patients with pleural space infection, including fever, cough, malaise, dyspnea, and pleuritic chest pain, closely resemble pneumonia and uncomplicated parapneumonic effusions. Thus, maintaining a high level of suspicion and ensuring a prompt, accurate diagnosis and management are crucial for favorable patient outcomes. With the rapid nuances in this field, this review provides a detailed overview of parapneumonic effusions and empyema, summarizing the literature on definition and classification, epidemiology, pathophysiology, diagnostic evaluation, treatment modalities, and prognostic factors of this diverse range of pleural diseases. Figure 1 summarizes a clinical approach to evaluation and management, and each section will be discussed in further detail in the text. 

## 2. Methods

PubMed and Google Scholar databases were searched for articles from 1970 through 1 April 2024, using search terms for complicated pleural effusions, pleural infections, empyema, and parapneumonic effusion. After carefully evaluating the abstracts, articles containing advances in clinical evaluation, diagnosis and treatment were then accessed and evaluated by the authors of this study. Articles were selected for inclusion based on relevance to day-to-day clinical practice with emphasis on groundbreaking studies as reflected in international guidelines. Randomized clinical trials, large longitudinal observational studies, and more recent articles were prioritized. Bibliographies of retrieved articles were analyzed for other relevant articles. 

## 3. Definitions

Parapneumonic effusions are pleural fluid collections that usually arise as a consequence of pneumonia and the ensuing pleural inflammatory response. These effusions are traditionally categorized into uncomplicated, complicated, and empyema [11]. The term “pleural infection” has also been suggested as a more comprehensive term, as about 30% of pleural infections have no previously identified pneumonia [12]. 

Uncomplicated parapneumonic effusions are characterized by free-flowing inflammatory sterile effusions within the pleural space, while complicated effusions indicate bacterial invasion and a more severe inflammatory response. Empyema, derived from the Greek word “empyein” (meaning “to suppurate”), is characterized by the presence of purulent material within the pleural cavity [13]. The term “frank empyema” is often used as a reference to the observation of frank pus when draining the pleural space. The transition from a parapneumonic effusion to empyema underscores the dynamic and overlapping nature of these conditions as well as the lack of early recognition and treatment [6]. This progression is accompanied by an array of clinical manifestations, including fever, chest pain, and respiratory distress.

## 4. Epidemiology

Given the heterogeneity in the definition of infectious pleural effusions, estimates of incidence and prevalence vary widely based on the parameters applied. A population-based study in France found an annual incidence of complicated parapneumonic effusions and empyema of approximately 2.5 cases per 100,000 adults, representing nearly 20% of all community-acquired pneumonia cases [14]. On the contrary, up to 41% of patients who were hospitalized with pneumonia developed a parapneumonic effusion, of which 5–10% progressed to complicated empyema [15]. Regardless of the advances in therapy, Mummadi et al. described a 37.5% relative increase in pleural infection-related hospitalization between 2007 and 2016 in the United States [16]. Substantial financial burdens are incurred when caring for those with infectious pleural effusions. Hospital, pharmacy, and radiologic imaging account for significant encountered [17]. Microbial culprits reflect the common etiologies of pneumonia, with Streptococcus pneumoniae being the most common offending organism isolated in empyema, though the prevalence of drug-resistant Staphylococcus aureus is increasing [18]. It is noteworthy to mention that recent studies have revealed that pleural fluid acts as a rich medium to support the growth of all relevant Streptococcus pneumoniae strains; furthermore, such culprits have been reported to be much better adapted to grow in pleural fluid than most other common respiratory pathogens, perhaps reflecting the epidemiological predominance [19]. Interestingly, pleural isolates from the subtopical region were reported to have a higher incidence of Gram-negative organisms in comparison to either of the topical regions [20]. Notably, not all infectious effusions are parapneumonic and, in such cases, the organisms observed in the pleural space are not the same as those observed in lung parenchyma infections indicating different spread patterns [12]. It has been reported that up to 5% of transudative effusions become complicated by secondary infections [21]. 

## 5. Pathophysiology

The pleural cavity is a closed system composed of two pleural layers, visceral and parietal. The normal pleural fluid production and absorption depends on the hydrostatic and oncotic pressure gradients across the two pleural layers and the pleural space and is principally mediated by the parietal pleura since it has higher hydrostatic pressures (fluid production) and contains the lymphatic stomata responsible for fluid resorption [2]. In the normal state, the pleural space contains 0.26 mL/kg of body weight of pleural fluid [22]. 

Parapneumonic effusions arise when the inflammatory reaction to pulmonary infection spreads across the visceral pleura into the pleural space, evoking fluid exudation rich in neutrophils and proteins through increased capillary permeability mediated by interleukin 8 and tumor necrosis factor-alpha. The progression of parapneumonic effusions to empyema represents a continuum involving three phases, described as exudative, fibrinopurulent, and organizing phases [23]. The evolution from exudative to fibrinopurulent is mediated by dysregulated cytokine expression and polymorphonuclear leukocytes increment in the pleural fluid leading to fibrin deposition [24]. As the infectious and inflammatory phases progress, fibrin clots and fibrin membrane deposition in the pleural cavity ensues within 5 to 10 days, leading to fluid loculations [25]. If left untreated, fibroblastic cells transform the fibrin deposits into a thick nonelastic pleural peel, resulting in what is known as a trapped lung, which is manifested as restrictive respiratory dysfunction [26]. Bacterial invasion may then induce empyema, characterized by frank pus in the pleural cavity, pneumatoceles, and pleural thickening or septations on imaging [3]. 

## 6. Diagnostic Evaluation

The diagnostic approach to complicated pleural effusions relies on a combination of imaging, fluid analysis, and tissue sampling. Ultrasound enables the accessible, real-time visualization of pleural fluid that can be used to safely guide thoracentesis, with minimum detection thresholds around 5–10 mL [27]. However, its utility is operator-dependent and limited for assessing underlying lung parenchyma distal to the effusion and debris. Chest radiography has been historically used as initial screening given its wide availability, though it requires approximately 150 mL of fluid before effusions become apparent [27]. Computed tomography (CT) scanning optimally defines pleural fluid contours, reveals accompanying lung pathologies, and identifies morphological features suggesting complicated effusions. CT scans can detect effusions as small as 3–5 mL [28]. The thickening of the pleural membrane can reflect pleural disease, and other features that suggest pleural infections include oblong configuration, split pleural signs, hypertrophy, and the increased density of the extra-pleural fat [29]. When further comparing those modalities, pleural ultrasound had a sensitivity of 69.2% (95% confidence interval (CI) 48.2–85.7%) and a specificity of 90.0% (CI 76.3–97.2%). Chest CT had a sensitivity of 76.9% (CI 56.3–91.0%) and a specificity of 65.0% (CI 48.3–79.4%), thus, making ultrasound a superior modality to rule in complicated pleural effusions. Notably, chest X-rays had a lower sensitivity of 61.5% (CI 40.6–79.8%) and a specificity of 60.0% (CI 43.3–75.1%) when compared to either modality [30]. It is noteworthy to mention that lateral decubitus X-ray increases the detection threshold of effusions. Nonetheless, the role of X-ray in diagnosing pleural effusions is limited in the era of the point-of-care ultrasound (POCUS), as its diagnostic accuracy is still high even when performed by physicians with less POCUS training [31]. Important characteristics indicating complicated parapneumonic effusions include echogenic swirling (which implies strong exudate or pus) and fibrin strands, visualized as septations or fully enclosed loculations [32]. 

The use of ultrasound to obtain pleural fluid samples has been supported by international guidelines for over two decades, as it markedly increased diagnostic yield with a lower risk of pneumothorax over blind sampling [33]. Furthermore, the use of ultrasound during thoracentesis can identify septations, which can indicate the inadequate evacuation of pleural effusions and the need for further therapy. Furthermore, their presence raises concerns for possible differences in pH between different fluid locules. Clinically significant pH variations lead to diagnostic challenges [34]. 

Further modalities such as PET scans and MRI can image the pleura; however, they have a limited role in the evaluation of infectious effusions. MRI imaging usually reveals low signal on T1-weighted and high signal on T2-weighted in infectious effusions. It is superior to CT in detecting pleural fluid septation and extra-pleural fat changes [29]. Further cytological analysis, cell counts with differentials, pH, glucose, and microbial stains, and cultures are often obtained to aid in classifying effusions. Pleural fluid PH below 7.20, glucose levels below 60 mg/dl, or lactate dehydrogenase that is more than three times the upper limit of serum (or above 1000 U/L) remain the gold standard tests to differentiate between complicated and uncomplicated parapneumonic effusions [Table 1 summarizes such findings] [11].

In cases of suspected pleural infection, microbiological confirmation is recommended to guide antibiotic therapy given the rising prevalence of drug-resistant organisms [35]. The yield from standard pleural culture was only 56% in a recently published systemic review of over 10,000 patients; however, rates improve when fluid is inoculated into blood culture bottles [20]. Culture positivity was not independently associated with mortality [36]. Medical thoracoscopy remains the gold standard modality of diagnosis, enabling direct visual sampling. Closed pleural biopsies exhibit comparable diagnostic yields; however, their utility in clinical practice centers around confirming an etiological diagnosis of exudative pleural effusion, particularly when malignancy is suspected [37]. When differentiating underlying etiologies remains challenging after fluid analysis and closed biopsy, video-assisted thoracoscopic (VATS) or open surgical lung biopsy may provide a definitive diagnosis through extensive pleural visualization and large tissue sampling [38]. Studies have assessed a wide array of pleural markers to aid in the diagnosis, differentiation, and progression of parapneumonic pleural effusions. For instance, pleural fluid CRP has been thought of as a sensitive marker to discriminate between complicated and uncomplicated parapneumonic effusions; however, its use has not been universally implemented [39]. Factors such as Nicotinamide phosphoribosyltransferase (NAMPT), L-36γ, and pleural cytokines have been shown to improve diagnostic accuracy; however, their availability is limited to specialized centers [40,41,42,43]. Regardless, this diagnostic field is rapidly progressing and multiple other markers such as the carcinoembryonic antigen, vascular endothelial growth factor A, programmed death-ligand 1, neutrophil gelatinase-associated lipocalin, the triggering receptor expressed in myeloid cells type-1, gamma-interferon, and calprotectin were evaluated but did not demonstrate advantages over the classic parameters [44]. 

## 7. Management

The management of complicated pleural effusions revolves around treating the underlying etiology and directly removing infected fluid when indicated. Uncomplicated parapneumonic effusions may resolve with antibiotics alone, while complicated parapneumonic effusions and empyemas often require drainage. The American Association for Thoracic Surgery consensus guidelines suggest that diagnostic thoracentesis should be performed on all parapneumonic effusions with a pleural fluid thickness of >1 cm on a chest X-ray or >2 cm on a chest CT [9]. Multiple modalities of drainage can be utilized, such as scheduled therapeutic thoracentesis, small-bore or large-bore chest tubes, video-assisted thoracoscopic surgery (VATS), and open decortication. The most recent British Thoracic Society Guideline for Pleural Disease discusses an algorithmic approach to pursuing thoracostomy. Immediate thoracostomy is pursued if frank pus is observed upon thoracentesis. Otherwise, immediate pH analysis should follow. Thoracostomies are recommended if pleural fluid pH < 7.20 or pH > 7.2 and <7.4 has an LDH > 900 IU/L pleural or fluid glucose of <40 mg/dL [10]. Empiric intravenous antibiotic therapy should be initiated once the diagnosis is known or suspected and should not be delayed while awaiting diagnostic procedures. Most antibiotics provide appropriate pleural penetration except for aminoglycosides [3]. Initial microbial coverage should target Streptococci, anaerobic bacteria if there is a suspicion of underlying aspiration, and Methicillin-resistant Staphylococcus aureus (MRSA) and Pseudomonas when hospital-acquired infections are suspected. The Thoracic Surgery guidelines recommend using a parenteral second- or third-generation cephalosporin (e.g., ceftriaxone) with metronidazole or parenteral aminopenicillin with β-lactamase inhibitor (e.g., ampicillin/sulbactam) for community-acquired infections. While using vancomycin, cefepime, and metronidazole or vancomycin and piperacillin/tazobactam (dosed for activity against *P. aeruginosa*) for hospital-acquired infections [9]. It is recommended to continue anaerobic coverage even in situations where an anaerobic organism is not identified on microbiological tests, due to the difficulties faced in culturing these organisms which commonly infect the pleural space [23]. 

Similar antibiotic regimens are used in those who develop pleural infections with an indwelling pleural catheter in place. Staphylococcus aureus is the most common pathogen and is observed in about half of the cases. Other common agents include *Pseudomonas aeruginosa* and Enterobacteriaceae [45]. Generally, catheters are kept in place whenever an infection is suspected. However, the catheter should be removed if there is failure in improvement with antibiotic therapy [6]. 

The optimal duration of treatment remains indeterminate. Physicians’ expertise, clinical condition, comorbidities as well as response to treatment usually dictate duration; however, most patients receive 2–6 weeks of antibiotics [9]. A common approach has been an initial intravenous course of antibiotics of 5–7 days to dampen the initial systemic inflammatory response and then transition to oral antibiotics [46,47]. A recently published trial assessing antibiotic duration in low- and medium-risk pleural infections compared a longer therapy duration of 34.5 days to a shorter duration of 20.5 days. Treatment failure occurred in 16.7% of patients in the short-course group and 12.5% of patients in the long-course group. In the intention-to-treat analysis, the odds ratio for treatment failure in the long-course group was 0.714 (95% CI 0.142–3.600, *p* = 0.683) [48]. The ODAPE trial was a noninferiority double-blind randomized control trial assessing a 2-week versus 3-week antibiotic strategy, and it showed excellent success rates in the group treated with a 2-week course, provided successful drainage and clinical stability had been achieved [49]. However, both these trials had small sample sizes of 50 and 55 patients, respectively, which calls for more high-quality methodological evidence prior to implementing shorter course antibiotic treatment. 

The utilization of intrapleural enzymatic therapy (IET) in patients who underwent tube thoracostomies has gained significant traction over the past decade. Combination therapy with fibrinolytics and endonucleases such as tissue plasminogen activator (tPA) and deoxyribonuclease (DNAse) administered through pleural catheters helps break down loculations to decrease viscosity and increase drainage [50]. The introduction of IET led to a paradigm shift in the treatment of pleural infections, as treatment success rates exceeded 90% while the failure rate of tube thoracostomy prior to the era of IET ranged from 9.4% to 48% [51,52]. Significant morbidity benefits were observed with the introduction of IET, for instance, length of hospital stay decreased by an average of 6.7 days (95% CI, −12.0 to −1.9) as did the frequency of surgical referral (odds ratio of 0.17, 95% CI 0.03 to 0.87) [50]. More recently, Dr. Bedawi et al. conducted a multicenter randomized clinical trial comparing IET to early VATS, and outcomes were in favor of IET as length of hospital stay and readmission rates were similar when the two groups were compared. Furthermore, compared with VATS, IET demonstrated a larger improvement in health-related quality of life as measured by EuroQol five-dimension health utility index [53]. Optimal dosing has not been established yet; however, the currently used regimen is derived from the original MIST 2 trial in which patients received a total of 10 mg of tPA and 5 mg of DNase intrapleurally twice a day in six doses [50]. However, such dosing might not be optimal in all patients [54]. An international survey of 49 practicing physicians involved in the management of pleural infections and who were either actively involved in pleural research and publications or were members of relevant pleural disease guideline panels observed a large variation in treatments offered [55]. The daily dosing of IET has been retrospectively studied with the results suggesting the safety and practicality of once-a-day dosing. However, the findings also suggested a higher surgical referral rate to what has been previously reported in the literature [56,57]. Furthermore, a study by Cheong et al. revealed that a modified regimen of 16 mg of tPA and 5 mg of DNase can be safe and effective. Nonetheless, the study lacked a comparative arm to those receiving the standard regimen [58]. Moreover, lower dose regimens have also been studied in smaller retrospective and observational studies. Despite the lack of more robust clinical guidelines, decreasing the dose of tPA to 5 mg also appeared to be safe and effective [59]. However, a large multicenter retrospective study of 1851 patients did not show a significant decrease in bleeding risk, therefore bringing into question the clinical utility of a lower dose regimen [60]. A randomized clinical trial assessing lower dose IET was initiated on April of 2024 and is expected to be complete by 2027 (Clinical Trial Identifier NCT05766124). On the other hand, the sequential administration of tPA and DNase is thought to be pharmacologically safer than concurrent administration. However, one study revealed no statistically significant difference between sequential or concurrent regimens in terms of the volume of pleural fluid drained or adverse effects [61]. Safe practice has been to hold systemic anticoagulation prior to administering IET as their use is associated with a 4.1% risk of pleural hemorrhage [50,60]. Other notable adverse effects of IET include pain and allergic reactions. The RAPID score has been shown to be a predictive marker in intrapleural hemorrhage after receiving the IET [60]. The utility of the RAPID score will be discussed in greater detail later in the text.

For decades, clinicians have been taught that the sun should never set on a complicated parapneumonic effusion, indicating the importance of drainage. However, the optimal size of chest tubes has been an area of debate, it was thought that larger-bore chest tubes are superior as smaller-sized ones are more likely to be occluded. However, it has been demonstrated that small bore (12–14 F) chest tubes are efficient as a first-line intervention, as analysis of varying sizes (from <10 F to >20 F) showed no difference in death, need for thoracic surgery, length of hospital stay, chest radiograph appearance, or lung function at three months [62,63]. Additionally, patient-reported outcomes were not in favor of large-bore chest drains due to the increased pain. The optimum timing of the chest tube removal depends on patients’ clinical condition, characteristics, and the volume of the draining fluid. Experts recommend that tubes should be left in place until the fluid becomes clear yellow and non-purulent with volume thresholds ranging from 200 mL per day to 500 mL per day [64]. It is recommended to remove chest tubes at the end of the expiration phase during the Valsalva maneuver [65]. 

A nationwide epidemiological analysis of pleural infections revealed that 64.9% of patients who underwent management with tube thoracostomy did not require a second therapeutic intervention [66]. However, surgical candidacy should be explored if patients are persistently septic despite chest tube drainage and antibiotics or if there is failure of chest tube drainage, antibiotics, and fibrinolytics [67]. Overall, surgical treatment is pursued in 36–65% of patients [6]. Historically, open thoracotomy was the surgical treatment of choice. In the current era, VATS decortication is preferred given similar results in terms of improvement, and superior outcomes in terms of decreased length of stay, morbidity, operative time, post-operative pain and air leaking, duration of chest drainage, and early return to daily activities [68]. The success rate of VATS is reported to range between 82–92% [69]. In the General Thoracic Surgery Database, complications occurred in 2875 patients (39.3%) and major morbidity occurred in 1138 patients (15.6%) undergoing VATS. Mortality was reported in 3.1% of patients [70]. Open thoracotomy is performed when a more complete decortication is required. A metanalysis performed by Pan et al. revealed that the rate of conversion of VATS to open thoracotomy to range between 8.7% to 59%, with factors such as delayed referral, Gram-negative bacteria, and surgeon experience often affecting conversion rates [71]. 

## 8. Emerging Therapies

The European Respiratory Society and the European Society of Thoracic Surgeons discuss the use of intrapleural antibiotics in their latest guidelines [53]. Direct pleural irrigation with antibiotics may have the theoretical advantage of reducing systemic side effects and antibiotic resistance. In current practice, intrapleural antibiotics are reserved for managing post-lung resection pleural infections [72]. On the other hand, intrapleural saline irrigation has been shown to yield superior resolution of CT pleural fluid volume and reduce surgical referrals and no randomized trials exist to this day; however, its use might be considered on a case-by-case basis if IET are contraindicated or there is a higher risk for bleeding [73]. 

Steroid therapy has been suggested as an additional treatment to help control the inflammatory response and halt the progression of parapneumonic effusion. The STOPPE trial compared dexamethasone to the placebo but did not show any improvement in terms of the quicker normalization of vital signs, improvement in inflammatory markers, or changes in radiographic pleural opacification. Steroid associated adverse effects were observed in the dexamethasone arm [74]. 

## 9. Prognosis and Outcomes

The overall healthcare burden associated with intrapleural infections is significant. It has been reported that 25% of patients require prolonged hospital stays of longer than 1 month, with a median hospital stay of 12–15 days [75]. In those undergoing VATS decortication, the median post-operative length of stay was 7 days.

The RAPID score, derived from the MIST 1 trial, is a prognostic score used to predict 3-month mortality [76]. It calculates baseline serum urea, patient age, pleural fluid purulence, infection source (community-acquired infection versus hospital-acquired infection), and serum albumin. The score categorizes patients into low-risk (RAPID score 0–2), medium-risk (RAPID score 3–4), and high-risk (RAPID score 5–7) groups, which were associated with mortality rates at 3 months of 3%, 9%, and 31%, respectively, in a prospective validation trial [75]. Overall mortality rates attributable to infectious pleural effusions range from 10–50%, with empyema conferring the highest mortality risk [77,78]. Low pleural fluid pH, high sepsis biomarker levels, and the need for surgical decortication predict worse outcomes [79]. There has been a substantial decrease in healthcare utilization, including inpatient use, length of stay, and costs over the past decade. Furthermore, inpatient mortality also decreased [67]. Thereafter, patient counseling on realistic prognosis based on clinical factors and response to initial interventions ensures aligned expectations and goals of care.

## 10. Areas for Future Research 

Multiple knowledge gaps persist despite the rapid evolvement in this field. The evaluation of host factors contributing to unfavorable outcomes would provide significant insight into appropriate management strategies and whether expedited surgical referral is required. Diagnostic uncertainty and low microbiological yields require the further evaluation of pleural sepsis biomarkers and studying modalities such as multiplex PCR and 16S rRNA gene sequencing. Additionally, investigating the role of viruses in pleural infections might lead to therapeutic advancements.

As far as potential research opportunities in treatment options are concerned, evaluating the optimal dosing and schedule for IET is of utmost significance. Methodologically sound, high-quality studies are needed to dictate future care due to the variability of practice patterns once it comes to patients with a higher risk of bleeding. Additionally, little is known regarding the longer-term outcomes of pleural infections beyond 12 months. Such data would provide useful insight into long-term care planning.

Lastly, the risk stratification of patients according to the RAPID score can potentially raise the opportunity for risk group-specific tailored treatment. Specific research questions can include whether repeat thoracentesis safely substitutes tube thoracostomies and the outpatient management of low-risk patients.

## 11. Conclusions 

Infectious pleural effusions carry significant mortality and morbidity burdens. Evaluation often starts with ultrasound due to its enhanced sensitivity and specificity when compared to chest X-Rays. Source control, obtained through the drainage of the pleural space, is of paramount importance along with antibiotic treatment. The introduction of IET over the past decade has led to a paradigm shift in treatment further translating into a decrease in healthcare utilization, surgical referrals, and overall mortality. However, further research is needed to enhance diagnostic and therapeutic modalities. 

## Figures and Tables

**Table 1:** Characteristics of parapneumonic pleural effusions and empyema. Notably, the clinical spectrum is heterogenous, and clinical overlap often exists. LDH = lactate dehydrogenase. Adapted from Sorino et al. [6], Park et al. [11], and Mercer et al. [34].

	Simple Parapneumonic Effusion	Complicated Parapneumonic Effusion	Empyema
Gross appearance	Clear fluid	Clear fluid or turbid	Purulent
Radiographical appearance	Free flowing	Septations and loculations	Septations, loculations, pleural thickening, and split pleura sign on CT
PH	>7.2	<7.2	<7.2
Glucose g/dL	>60	<60	<60
LDH U/L	<1000	>1000	>1000
Fluid culture	No growth	No growth	Positive growth
Treatment	Antibiotics	Antibiotics ± drainage	Antibiotics and drainage

## References

[1] Tian P., Qiu R., Wang M., Xu S., Cao L., Yang P., Li W. (2021). Prevalence, Causes, and Health Care Burden of Pleural Effusions Among Hospitalized Adults in China. JAMA Netw. Open.

[2] Karpathiou G., Péoc’h M., Sundaralingam A., Rahman N., Froudarakis M.E. (2022). Inflammation of the Pleural Cavity: A Review on Pathogenesis, Diagnosis and Implications in Tumor Pathophysiology. Cancers.

[3] Hassan M., Patel S., Sadaka A.S., O Bedawi E., Corcoran J.P., Porcel J.M. (2021). Recent Insights into the Management of Pleural Infection. Int. J. Gen. Med..

[4] Bhatnagar R., Corcoran J.P., Maldonado F., Feller-Kopman D., Janssen J., Astoul P., Rahman N.M. (2016). Advanced medical interventions in pleural disease. Eur. Respir. Rev..

[5] Wang S., Zhang R., Wan C., Qin J., Hu X., Shen Y., Chen L., Wen F. (2023). Incidence of complications from indwelling pleural catheter for pleural effusion: A meta-analysis. Clin. Transl. Sci..

[6] Sorino C., Mondoni M., Lococo F., Marchetti G., Feller-Kopman D. (2022). Optimizing the management of complicated pleural effusion: From intrapleural agents to surgery. Respir. Med..

[7] McNally E., Ross C., Gleeson L.E. (2023). The tuberculous pleural effusion. Breathe.

[8] Cohen L.A., Light R.W. (2015). Tuberculous Pleural Effusion. Turk. Thorac. J..

[9] Shen K.R., Bribriesco A., Crabtree T., Denlinger C., Eby J., Eiken P., Jones D.R., Keshavjee S., Maldonado F., Paul S. (2017). The American Association for Thoracic Surgery consensus guidelines for the management of empyema. J. Thorac. Cardiovasc. Surg..

[10] Roberts M.E., Rahman N.M., Maskell N.A., Bibby A.C., Blyth K.G., Corcoran J.P., Edey A., Evison M., de Fonseka D., Hallifax R. (2023). British Thoracic Society Guideline for pleural disease. Thorax.

[11] Park H.J., Choi C.M. (2020). Can parapneumonic effusion be diagnosed only with pleural fluid analysis?. J. Thorac. Dis..

[12] Brims F., Popowicz N., Rosenstengel A., Hart J., Yogendran A., Read C.A., Lee F., Shrestha R., Franke A., Lewis J.R. (2019). Bacteriology and clinical outcomes of patients with culture-positive pleural infection in Western Australia: A 6-year analysis. Respirology.

[13] Brims F.J.H., Lansley S.M., Waterer G.W., Lee Y.C.G. (2010). Empyema thoracis: New insights into an old disease. Eur. Respir. Rev..

[14] Ferreiro L., San José M.E., Valdés L. (2015). Management of Parapneumonic Pleural Effusion in Adults. Arch. Bronconeumol..

[15] Zhong M., Ni R., Zhang H., Sun Y. (2023). Analysis of clinical characteristics and risk factors of community-acquired pneumonia complicated by parapneumonic pleural effusion in elderly patients. BMC Pulm. Med..

[16] Mummadi S.R., Stoller J.K., Lopez R., Kailasam K., Gillespie C.T., Hahn P.Y. (2021). Epidemiology of Adult Pleural Disease in the United States. Chest.

[17] Shah S.S., Have T.R.T., Metlay J.P. (2010). Costs of treating children with complicated pneumonia: A comparison of primary video-assisted thoracoscopic surgery and chest tube placement. Pediatr. Pulmonol..

[18] Jovanovic D. (2021). Etiopathogenesis of malignant pleural effusion. AME Med. J..

[19] Popowicz N.D., Lansley S.M., Cheah H.M., Kay I.D., Carson C.F., Waterer G.W., Paton J.C., Brown J.S., Lee Y.C.G. (2017). Human pleural fluid is a potent growth medium for Streptococcus pneumoniae. PLoS ONE.

[20] Hassan M., Cargill T., Harriss E., Asciak R., Mercer R.M., Bedawi E.O., McCracken D.J., Psallidas I., Corcoran J.P., Rahman N.M. (2019). The microbiology of pleural infection in adults: A systematic review. Eur. Respir. J..

[21] Finley C., Clifton J., FitzGerald J.M., Yee J. (2008). Empyema: An increasing concern in Canada. Can. Respir. J..

[22] Noppen M., DE Waele M., Li R., Gucht K.V., D’Haese J., Gerlo E., Vincken W. (2000). Volume and cellular content of normal pleural fluid in humans examined by pleural lavage. Am. J. Respir. Crit. Care Med..

[23] Singh S.K., Singh S., Tentu A.K. (2019). Management of parapneumonic effusion and empyema. J. Assoc. Chest Physicians.

[24] Yang C.-Y., Kuo Y.-H., Chen M., Wang C.-L., Shih L.-J., Liu Y.-C., Hsueh P.-C., Lai Y.-H., Chu C.-M., Wu C.-C. (2021). Pleural cytokines MIF and MIP-3α as novel biomarkers for complicated parapneumonic effusions and empyema. Sci. Rep..

[25] Subotic D., Lardinois D., Hojski A. (2018). Minimally invasive thoracic surgery for empyema. Breathe.

[26] Komissarov A.A., Rahman N.M., Lee Y.C.G., Florova G., Shetty S., Idell R., Ikebe M., Das K., Tucker T.A., Idell S. (2018). Fibrin turnover and pleural organization: Bench to bedside. Am. J. Physiol. Cell Mol. Physiol..

[27] Wang T., Du G., Fang L., Bai Y., Liu Z., Wang L. (2022). Value of ultrasonography in determining the nature of pleural effusion: Analysis of 582 cases. Medicine.

[28] Heffner J.E., Klein J.S., Hampson C. (2010). Diagnostic utility and clinical application of imaging for pleural space infections. Chest.

[29] Hassan M., Touman A.A., Grabczak E.M., Skaarup S.H., Faber K., Blyth K.G., Pochepnia S. (2024). Imaging of pleural disease. Breathe.

[30] Svigals P.Z., Chopra A., Ravenel J.G., Nietert P.J., Huggins J.T. (2017). The accuracy of pleural ultrasonography in diagnosing complicated parapneumonic pleural effusions. Thorax.

[31] Zaki H.A., Albaroudi B., Shaban E.E., Shaban A., Elgassim M., Almarri N.D., Basharat K., Azad A.M. (2024). Advancement in pleura effusion diagnosis: A systematic review and meta-analysis of point-of-care ultrasound versus radiographic thoracic imaging. Ultrasound J..

[32] Marchetti G., Arondi S., Baglivo F., Lonni S., Quadri F., Valsecchi A., Venturoli N., Ceruti P. (2018). New insights in the use of pleural ultrasonography for diagnosis and treatment of pleural disease. Clin. Respir. J..

[33] Havelock T., Teoh R., Laws D., Gleeson F. (2010). Pleural procedures and thoracic ultrasound: British thoracic society pleural disease guideline 2010. Thorax.

[34] Mercer R.M., Corcoran J.P., Porcel J.M., Rahman N.M., Psallidas I. (2019). Interpreting pleural fluid results. Clin. Med..

[35] Elsheikh A., Bhatnagar M., Rahman N.M. (2023). Diagnosis and management of pleural infection. Breathe.

[36] Chan K.P., Ng S.S.S., Ling K.C., Ng K.C., Lo L.P., Yip W.H., Ngai J.C.L., To K.W., Ko F.W.S., Lee Y.C.G. (2023). Phenotyping empyema by pleural fluid culture results and macroscopic appearance: An 8-year retrospective study. ERJ Open Res..

[37] Zhang T., Wan B., Wang L.L., Li C., Xu Y., Wang X., Li H., Song Y., Lin D., Zhan P. (2020). The diagnostic yield of closed needle pleural biopsy in exudative pleural effusion: A retrospective 10-year study. Ann. Transl. Med..

[38] Sundaralingam A., Bedawi E.O., Rahman N.M. (2020). Diagnostics in Pleural Disease. Diagnostics.

[39] Kogan Y., Sabo E., Odeh M. (2020). Diagnostic Value of C-Reactive Protein in Discrimination between Uncomplicated and Complicated Parapneumonic Effusion. Diagnostics.

[40] Huang J., Guo L., Kang H.-W., Lv D., Lin W., Li C.-F., Huang X.-Q., Ding Q.-L. (2021). Nicotinamide phosphoribosyltransferase as a biomarker for the diagnosis of infectious pleural effusions. Sci. Rep..

[41] Yan Z., Wen J.-X., Wang H., Jiang T.-W., Huang J.-H., Chen H., Yan L., Hu Z.-D., Zheng W.-Q. (2022). Diagnostic accuracy of pleural fluid lactate dehydrogenase to adenosine deaminase ratio for tuberculous pleural effusion: An analysis of two cohorts. BMC Pulm. Med..

[42] Arnold D.T., Hamilton F.W., Elvers K.T., Frankland S.W., Zahan-Evans N., Patole S., Medford A., Bhatnagar R., Maskell N.A. (2020). Pleural Fluid suPAR Levels Predict the Need for Invasive Management in Parapneumonic Effusions. Am. J. Respir. Crit. Care Med..

[43] Guo L., Zhang Q., Lv C., Ma X., Song X., Huang J., Chen W., Li C., Ding Q. (2023). A novel biomarker for pleural effusion diagnosis: Interleukin-36γ in pleural fluid. J. Clin. Lab. Anal..

[44] Faria D.K., Faria C.S., Doi D., Acencio M.M., Antonangelo L. (2019). Hybrid panel of biomarkers can be useful in the diagnosis of pleural and peritoneal effusions. Clin. Chim. Acta.

[45] Faiz S.A., Pathania P., Song J., Li L., Balachandran D.D., Ost D.E., Morice R.C., Shannon V.R., Bashoura L., Eapen G.A. (2017). Indwelling Pleural Catheters for Patients with Hematologic Malignancies. A 14-Year, Single-Center Experience. Ann. Am. Thorac. Soc..

[46] Iversen K., Ihlemann N., Gill S.U., Madsen T., Elming H., Jensen K.T., Bruun N.E., Høfsten D.E., Fursted K., Christensen J.J. (2019). Partial Oral versus Intravenous Antibiotic Treatment of Endocarditis. N. Engl. J. Med..

[47] Li H.-K., Rombach I., Zambellas R., Walker A.S., McNally M.A., Atkins B.L., Lipsky B.A., Hughes H.C., Bose D., Kümin M. (2019). Oral versus Intravenous Antibiotics for Bone and Joint Infection. N. Engl. J. Med..

[48] Hassan M., Gad-Allah M., El-Shaarawy B., El-Shazly A.M., Daneshvar C., Sadaka A.S. (2023). The Short versus Long Antibiotic Course for Pleural Infection Management (SLIM) randomised controlled open-label trial. ERJ Open Res..

[49] Porcel J.M., Ferreiro L., Rumi L., Espino-Paisan E., Civit C., Pardina M., Schoenenberger-Arnaiz J.A., Valdes L., Bielsa S. (2020). Two vs. three weeks of treatment with amoxicillin-clavulanate for stabilized community-acquired complicated parapneumonic effusions. A preliminary non-inferiority, double-blind, randomized, controlled trial. Pleura Peritoneum.

[50] Rahman N.M., Maskell N.A., West A., Teoh R., Arnold A., Mackinlay C., Peckham D., Davies C.W., Ali N., Kinnear W. (2011). Intrapleural use of tissue plasminogen activator and dnase in pleural infection. N. Engl. J. Med..

[51] Igbokwe J., Okidi O., Aja A. (2010). Management of Pleural Fluid Collection with Tube Thoracostomy in a Tertiary Health Facility in Abakaliki, Nigeria. Ebonyi Med. J..

[52] Majid A., Kheir F., Folch A., Fernandez-Bussy S., Chatterji S., Maskey A., Fashjian M., Cheng G., Ochoa S., Alape D. (2016). Concurrent Intrapleural Instillation of Tissue Plasminogen Activator and DNase for Pleural Infection. A Single-Center Experience. Ann. Am. Thorac. Soc..

[53] Bedawi E.O., Ricciardi S., Hassan M., Gooseman M.R., Asciak R., Castro-Añón O., Armbruster K., Bonifazi M., Poole S., Harris E.K. (2022). ERS/ESTS statement on the management of pleural infection in adults. Eur. Respir. J..

[54] Pathak V., Adhikari L., Zhou C. (2023). Effects of Concurrent Dosing on the Efficacy of Tissue Plasminogen Activator and Deoxyribonuclease in the Treatment of Pleural Infection. Cureus.

[55] Lau E.P.M., Eshraghi M., Dootson K., Yeoh C., Phu W.Y., Lee Y.C.G., Popowicz N.D. (2021). An international survey on the use of intrapleural tissue plasminogen activator/DNase therapy for pleural infection. ERJ Open Res..

[56] Mehta H.J., Biswas A., Penley A.M., Cope J., Barnes M., Jantz M.A. (2016). Management of Intrapleural Sepsis with Once Daily Use of Tissue Plasminogen Activator and Deoxyribonuclease. Respiration.

[57] Smith D., Shaw H., Ryder T. (2023). Intrapleural tissue plasminogen activator and deoxyribonuclease administered concurrently and once daily for complex parapneumonic pleural effusion and empyema. Intern. Med. J..

[58] Cheong X.K., Ban A.Y.-L., Ng B.H., Abeed N.N.N., Ismail N.A.N., Fuad N.F.N., Zakaria S.Z.S., Ghan S.L., Hamid M.F.A. (2022). Modified regimen intrapleural alteplase with pulmozyme in pleural infection management: A tertiary teaching hospital experience. BMC Pulm. Med..

[59] Popowicz N., Bintcliffe O., De Fonseka D., Blyth K.G., Smith N.A., Piccolo F., Martin G., Wong D., Edey A., Maskell N. (2017). Dose De-escalation of Intrapleural Tissue Plasminogen Activator Therapy for Pleural Infection. The Alteplase Dose Assessment for Pleural Infection Therapy Project. Ann. Am. Thorac. Soc..

[60] Akulian J., Bedawi E.O., Abbas H., Argento C., Arnold D.T., Balwan A., Batra H., Becerra J.P.U., Belanger A., Berger K. (2022). Bleeding Risk with Combination Intrapleural Fibrinolytic and Enzyme Therapy in Pleural Infection: An International, Multicenter, Retrospective Cohort Study. Chest.

[61] Kheir F., Cheng G., Rivera E., Folch A., Folch E., Fernandez-Bussy S., Keyes C., Parikh M., Channick C., Chee A. (2018). Concurrent Versus Sequential Intrapleural Instillation of Tissue Plasminogen Activator and Deoxyribonuclease for Pleural Infection. J. Bronchol. Interv. Pulmonol..

[62] Rahman N.M., Maskell N.A., Davies C.W., Hedley E.L., Nunn A.J., Gleeson F.V., Davies R.J. (2010). The relationship between chest tube size and clinical outcome in pleural infection. Chest.

[63] Mehra S., Heraganahally S., Sajkov D., Morton S., Bowden J. (2020). The effectiveness of small-bore intercostal catheters versus large-bore chest tubes in the management of pleural disease with the systematic review of literature. Lung India.

[64] Anderson D., Chen S.A., Godoy L.A., Brown L.M., Cooke D.T. (2022). Comprehensive Review of Chest Tube Management A Review. JAMA Surg..

[65] Novoa N.M., Jiménez M.F., Varela G. (2017). When to Remove a Chest Tube. Thorac. Surg. Clin..

[66] Gupta I., Eid S.M., Gillaspie E.A., Broderick S., Shafiq M. (2021). Epidemiologic Trends in Pleural Infection. A Nationwide Analysis. Ann. Am. Thorac. Soc..

[67] Santoshi R.K., Chandar P., Gupta S.S., Kupfer Y., Wiesel O. (2022). From Chest Wall Resection to Medical Management: The Continued Saga of Parapneumonic Effusion Management and Future Directions. Cureus.

[68] Ricciardi S., Giovanniello D., Carleo F., Di Martino M., Jaus M.O., Mantovani S., Treggiari S., Tritapepe L., Cardillo G. (2023). Which Surgery for Stage II–III Empyema Patients? Observational Single-Center Cohort Study of 719 Consecutive Patients. J. Clin. Med..

[69] Höfken H., Herrmann D., Ewig S., Volmerig J., Hecker E. (2018). Video-Assisted Thoracoscopic Surgery of Parapneumonic Empyema—A 10-year Single-Centre Experience. Pneumologie.

[70] DeBiasi E.M., Pisani M.A., Murphy T.E., Araujo K., Kookoolis A., Argento A.C., Puchalski J. (2015). Mortality among Patients with Pleural Effusion Undergoing Thoracentesis. Eur. Respir. J..

[71] Pan H., He J., Shen J., Jiang L., Liang W., He J. (2017). A meta-analysis of video-assisted thoracoscopic decortication versus open thoracotomy decortication for patients with empyema. J. Thorac. Dis..

[72] Bhatnagar R., Skouras V.S., Rahman N.M., Psallidas I. (2017). Antibiotics for pleural infections. ERS Monogr..

[73] Guinde J., Laroumagne S., Chollet B., Trias-Sabrià P., Dutau H., Astoul P. (2021). Saline lavage for the management of severe pleural empyema: A cohort study. Clin. Respir. J..

[74] Fitzgerald D.B., Waterer G.W., Budgeon C., Shrestha R., Fysh E.T., Muruganandan S., Stanley C., Saghaie T., Badiei A., Sidhu C. (2022). Steroid Therapy and Outcome of Parapneumonic Pleural Effusions (STOPPE) A Pilot Randomized Clinical Trial. Am. J. Respir. Crit. Care Med..

[75] Corcoran J.P., Psallidas I., Gerry S., Piccolo F., Koegelenberg C.F., Saba T., Daneshvar C., Fairbairn I., Heinink R., West A. (2020). Prospective validation of the RAPID clinical risk prediction score in adult patients with pleural infection: The PILOT study. Eur. Respir. J..

[76] Rahman N.M., Kahan B.C., Miller R.F., Gleeson F.V., Nunn A.J., Maskell N.A. (2014). A Clinical score (RAPID) to identify those at risk for poor outcome at presentation in patients with pleural infection. Chest.

[77] Touray S., Sood R.N., Lindstrom D., Holdorf J., Ahmad S., Knox D.B., Sosa A.F. (2018). Risk Stratification in Patients with Complicated Parapneumonic Effusions and Empyema Using the RAPID Score. Lung.

[78] Markatis E., Perlepe G., Afthinos A., Pagkratis K., Varsamas C., Chaini E., Papanikolaou I.C., Gourgoulianis K.I. (2022). Mortality among Hospitalized Patients with Pleural Effusions. A Multicenter, Observational, Prospective Study. Front. Med..

[79] Thomsen T.W., DeLaPena J., Setnik G.S. (2014). Diagnostic Approach to Pleural Effusion. Am. Fam. Physician.

